# Two New Cyototoxic Cardenolides from the Whole Plants of *Adonis multiflora* Nishikawa & Koki Ito

**DOI:** 10.3390/molecules201119722

**Published:** 2015-11-23

**Authors:** Jae-Woo Jung, Nam-In Baek, Jeon Hwang-Bo, Seung-Su Lee, Ji-Hae Park, Kyeong-Hwa Seo, Jung-Hwa Kwon, Eun-Ji Oh, Dae-Young Lee, In-Sik Chung, Myun-Ho Bang

**Affiliations:** 1 Graduate School of Biotechnology and Department of Oriental Medicine Biotechnology, Kyung Hee University, Yongin 446-701, Korea; jaewoo4848@naver.com (J.-W.J.); nibaek@khu.ac.kr (N.-I.B.); ldkhhghh@khu.ac.kr (S.-S.L.); wlgo3411@hanmail.net (J.-H.P.); kyeonghwaseo@khu.ac.kr (K.-H.S.); life-kjh@hanmail.net (J.-H.K.); jk3172@nate.com (E.-J.O.); 2 Graduate School of Biotechnology and Department of Genetic Engineering, Kyung Hee University, Yongin 446-701, Korea; hbj3286@khu.ac.kr (J.H.-B.); ischung@khu.ac.kr (I.-S.C.); 3 Department of Herbal Crop Research, National Institute of Horicultural and Herbal Science, RDA, Eumseong 369-873, Korea; dylee0809@korea.kr

**Keywords:** *Adonis multiflora*, adonioside A, adonioside B, cardenolide, cytotoxic activity

## Abstract

A phytochemical investigation of the whole plants of *Adonis multiflora* Nishikawa & Koki Ito. resulted in the isolation and identification of two new cardenolides—adonioside A (**1**) and adonioside B (**6**)—as well as four known cardenolides: tupichinolide (**2**) oleandrine (**3**), cryptostigmin II (**4**), and cymarin (**5**). Their structures were elucidated on the basis of NMR, MS, and IR spectroscopic analyses. Compounds **1**, **2**, **5**, and **6** showed significant cytotoxicity against six human cancer cell lines (HCT-116, HepG2, HeLa, SK-OV-3, and SK-MEL-5, and SK-BR-3).

## 1. Introduction

Cardenolides, a chemical class within the cardiac glycosides, have a five-membered lactone group in the β position at C17 [1]. The mechanisms of these compounds are known to inhibit Na^+^/K^+^-ATPase, activate the cation pump, and increase in intracellular calcium concentration through cellular output of Na^+^ and intake of K^+^ [2]. Because of these biological actions, cardenolides have been used in the treatment of heart failure [3]. In addition, many researchers have suggested that cardenolides may inhibit the growth of cancer cells, and have described them as anticancer agents with fewer side effects [4,5].

**Figure 1:**
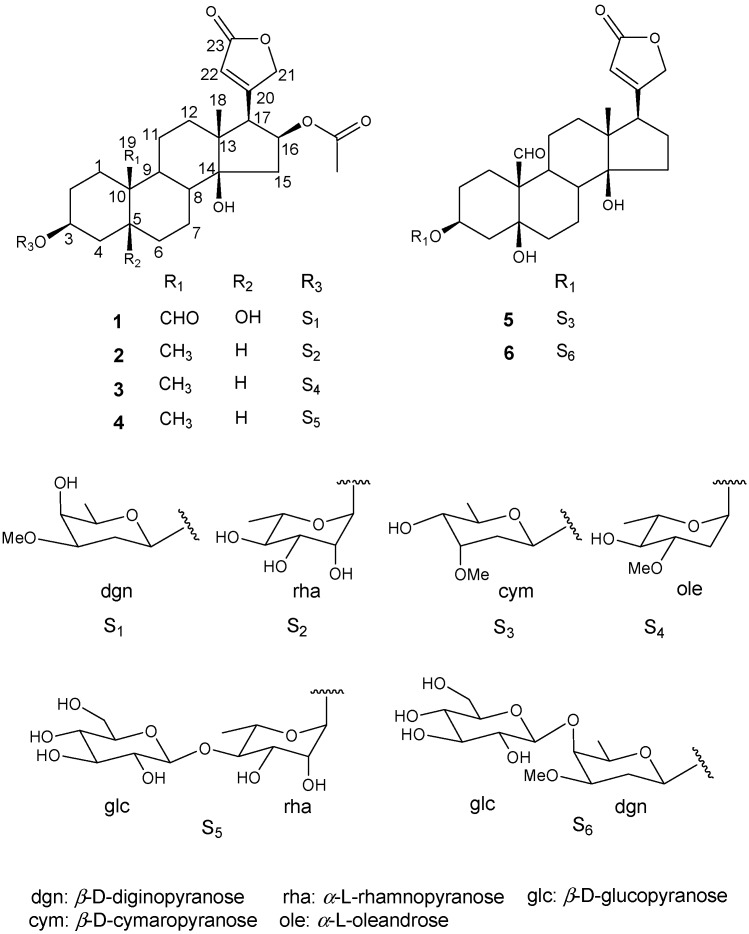
Compounds **1**–**6** isolated from the whole plants of *Adonis multiflora*.

Cardiac glycosides were isolated from several plant families of Ranunculaceae, Scrophulariazea, Apocynaceae, and Liliaceae, along with pregnane glycosides [6]. In Korea, the *Adonis* family is mainly comprised of three species, *A. amurensis*, *A. pseudoamurensis*, and *A. multiflora* based on RAPD analysis [7,8]. Previous phytochemical studies conducted on the roots of *A. amurensis*, the most well-known *Adonis* species, have identified several cardenolides: corchoroside A, covallatoxin, cymarin, cymarol, digitoxigenin 3-*O*-β-d-cymaroside, k-strophanthin, and k-strophanthin-β [9]. However, little has been reported concerning the biological and phytochemical properties of *A. multiflora*, except a brief report [10]. We have confirmed the presence of cardenolide spots in the TLC of ethanolic extracts from whole plants of *A. multiflora* based on the UV absorption pattern and the colors produced by spraying with a 10% H_2_SO_4_ solution and heating. Over the course of investigating cardenolides in whole plants of *A. multiflora* Nishikawa & Koki Ito, two new cardenolides **1** and **6** were identified and structurally determined, along with four known ones **2**–**5** (Figure 1). The cardenolides were then evaluated for cytotoxity against six human cancer cell lines (HCT-116, HepG2, HeLa, SK-OV-3, SK-BR-3, and SK-MEL-5).

## 2. Results and Discussion

The EtOH extracts were partitioned into CH_2_Cl_2_, EtOAc, BuOH, and H_2_O fractions. Repeated SiO_2_ and ODS column chromatography of the CH_2_Cl_2_ and BuOH fractions resulted in the identification of two new cardenolides, named adonioside A (**1**) and adonioside B (**6**), along with four known cardenolides **2**–**5**. The known compounds were identified as tupichinolide (**2**), oleandrine (**3**), cryptostigmin II (**4**), and cymarin (**5**) on the basis of spectroscopic analysis and the identities were confirmed by comparing their measured spectroscopic data with those reported in the literature [11,12,13,14].

Compound **1** was isolated as a white powder and showed IR absorbance bands representing OH (3384 cm^−1^), CHO (1737 cm^−1^), and C=C (1639 cm^−1^) groups. The molecular weight was determined to be 606 from the molecular ion peak *m/z* 605 [M − H]^−^ in the negative FAB-MS spectrum, and a molecular formula of C_32_H_46_O_11_ was determined from the high-resolved molecular ion peak ([M − H]^−^, *m*/*z* 605.2971, calc. for C_32_H_45_O_11_, 605.2962) in the negative HR-FAB-MS. The ^1^H-NMR spectrum (Table 1) exhibited the characteristics of an α,β-unsaturated-γ-lactone ring, with signals at δ(H) 5.95 (dd, *J* = 1.6, 1.6 Hz, H-22), 4.94 (dd, *J* = 18.4, 1.6 Hz, H-21_a_), and 4.83 (dd, *J* = 18.4, 1.6 Hz, H-21_b_) as well as a tertiary methyl signal at δ(H) 0.92 (s, H-18), a formyl signal at δ(H) 9.92 (s, H-19), two O-bearing CH signals at δ(H) 4.19 (br.s, H-3) and 5.41 (ddd, *J* = 9.6, 8.4, 8.4 Hz, H-16), and an AcO signal at δ(H) 1.95 (s, H-AcO), which suggested the presence of a cardenolide moiety with two oxygenated methines and an AcO group. In addition, a hemiacetal signal at δ(H) 4.50 (dd, *J* = 9.6, 2.4 Hz, H-1′), three O-bearing CH signals at δ(H) 3.31~3.67 (H-3′~5′), an O-bearing CH_3_ signal at δ(H) 3.36 (s, H-CH_3_O), a CH_2_ signal at δ(H) 2.05 (m, H-2′_a_) and 1.66 (m, H-2′_b_), and a CH_3_ signal at δ(H) 1.33 (d, *J* = 6.8 Hz, H-6′), indicated that **1** was a cardiac monoglycoside with a β-diginopyranoside.

The ^13^C-NMR spectrum showed 32 C-atoms signals (Table 1). The aglycone with α,β-unsaturated-γ-lactone ring signals observed at δ(C) 173.9 (C-23), 167.3 (C-20), 121.5 (C-22), and 75.5 (C-21), a formyl signal at δ(C) 208.1 (C-19), two O-bearing quaternary signals at δ(C) 73.5 (C-5) and 83.9 (C-14), AcO signals at δ(C) 170.4 (C-OAc) and 21.0 (C-OAc), two O-bearing CH signals at δ(C) 74.1 (C-3) and 73.7 (C-16), and a tertiary CH_3_ signal at δ(C) 15.8 (C-18) indicated that the aglycone was a cardenolide with four hydroxyls, one formyl, and one AcO group. The monosaccharide carbon signals, including a hemiacetal signal at δ(C) 98.9 (C-1′), three O-bearing CH signals at δ(C) 77.6 (C-3′), 70.7 (C-5′), 66.9 C-4′), a CH_3_O signal at δ(C) 55.8 (C-CH_3_O), a CH_2_ signal at δ(C) 31.5 (C-2′), and a CH_3_ signal at δ(C) 16.7 (C-6′), allowed us to conclude that the sugar was β-diginopyranose.

**Figure 2:**
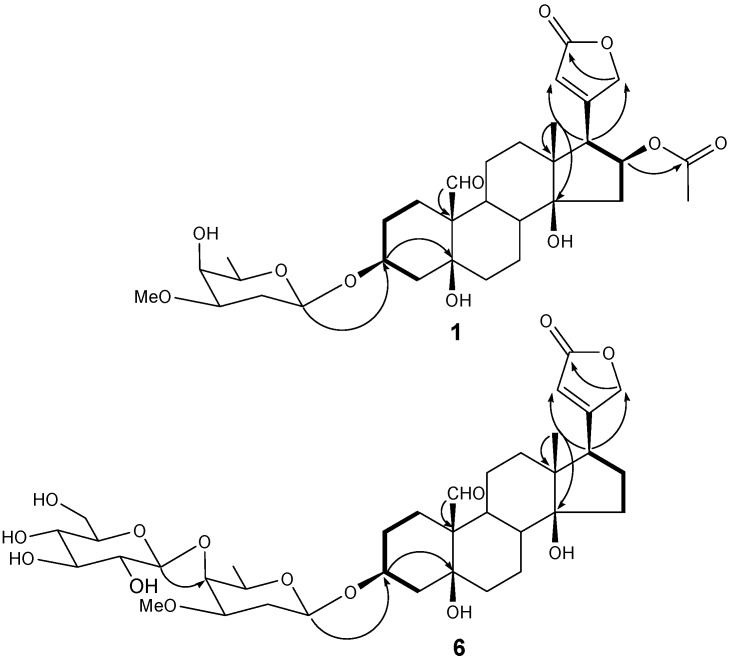
^1^H-^1^H-COSY (−) and gHMBC (H→C) key correlations of compounds **1** and **6**.

Acid hydrolysis of **1** and purification of the hydrolysate using column chromatography resulted in a sugar compound, which was identified to be a diginopyranose by direct comparison between its *R_f_* values on the SiO_2_ TLC (0.47 with CHCl_3_/MeOH 9:1, and 0.19 with CH_2_Cl_2_/EtOH 9:1) and those of an authentic sample. The specific rotation value of the obtained sugar (${\left[\text{α}\right]}_{D}^{20}$ = +56.8, *c* = 0.11, H_2_O), and the large *J* value of the anomeric signal at δ(H) 4.50 (dd, *J* = 9.6, 2.4 Hz, H-1′) revealed the sugar to be β-d-diginopyranose. The location of β-d-diginopyranose, methyl, formyl, hydroxyls, and AcO groups of **1** were determined from the connectivity between the oxygenated methine proton δ(H) 4.50 (1H, d, *J* = 9.6, 2.4 Hz, H-1′) and O-bearing CH carbon δ(C) 74.1 (C-3), tertiary methyl proton δ(H) 0.92 (s, H-18) and quaternary carbon δ(C) 49.8 (C-13), formyl proton δ(H) 9.92 (s, H-19) and quaternary carbon δ(C) 54.3 (C-10), tertiary methyl proton δ(H) 0.92 (s, H-18) and O-bearing quaternary carbon δ(C) 83.9 (C-14) and O-bearing methine proton δ(H) 5.41 (ddd, *J* = 9.6, 8.4, 8.4 Hz, H-16) and AcO carbon δ(C) 170.4 (C-OAc) in the HMBC spectrum, respectively. The location of the lactone group was deduced from the connectivity between the methylene protons H-15 δ(H) 2.59 (dd, *J* = 15.6, 9.6 Hz, H-15), O-bearing methine proton δ(H) 5.41 (ddd, *J* = 9.6, 8.4, 8.4 Hz, H-16) and the methine proton δ(H) 3.15 (d, *J* = 8.4 Hz, H-17) in the COSY spectrum (Figure 2). Taken together, compound **1** was determined to be a 16-β-acetoxystrophanthidin 3-*O*-β-d-digonopyranoside, a new cardenolide named adonioside A.

Compound **6** was also isolated as a white powder and showed IR absorbance bands of OH (3387 cm^−1^), CHO (1742 cm^−1^), and C=C (1647 cm^−1^) groups. The molecular weight was determined to be 710 due to the pseudomolecular ion peak *m/z* 733 [M + Na]^+^ in the positive FAB-MS spectrum, and the molecular formula of C_36_H_54_O_14_ was determined by the high-resolution pseudomolecular ion peak ([*M* + Na]^+^, *m*/*z* 733.3511, calc. for C_36_H_54_O_14_Na, 733.3411) in the positive HR-FAB-MS. The ^1^H-NMR spectrum (Table 1) displayed a formyl signal at δ(H) 10.33 (s, H-19), an olefin CH signal at δ(H) 6.10 (s, H-22), O-bearing CH_2_ signals at δ(H) 5.25 (d, *J* = 18.4 Hz, H-21_a_) and 4.99 (d, *J* = 18.4 Hz, H-21_b_), O-bearing CH signal at δ(H) 4.64 (br.s, H-3), and a tertiary CH_3_ at δ(H) 0.98 (s, H-18) indicating that **6** has a cardenolide skeleton. Also, two hemiacetal signals at δ(H) 5.07 (d, *J* = 7.6 Hz, H-1′) and 4.69 (br.d, *J* = 9.2 Hz, H-1′′) were observed, and their large *J* values confirmed that the anomer hydroxyls were in β form. Two hexoses were determined to be β-diginopyranosyl-(1→4)-β-diginopyranose through comparisons between ^13^C-NMR data and those reported in previously published literature [15]. Acid hydrolysis of **6** and comparison of the specific rotation values of two isolated sugars [**6a**: ${\left[\text{α}\right]}_{D}^{20}$ = +55.5 (*c* = 0.12, H_2_O), **6b**: ${\left[\text{α}\right]}_{D}^{20}$ = +49.3 (*c* = 0.12, H_2_O)] led to the identification of two sugars, d-diginopyranose (${\left[\text{α}\right]}_{D}^{20}$ = +59.6) and d-glucopyranose (${\left[\text{α}\right]}_{D}^{20}$ = +52.5) [16,17]. The locations of functional groups were determined by gCOSY and gHMBC experiments (Figure 2). Thus, compound **6** was identified as strophanthidin 3-*O*-β-d-diginopyranosyl-(1→4)-β-d-glucopyronoside, a new cardenolide named adonioside B.

**Table 1:** ^1^H- and ^13^C-NMR Data (400 and 100 MHz, resp.) of compounds **1** and **6**.

Position	1 (CD_3_OD)		6 (C_5_D_5_N)	
	δ(H)	δ(C)	δ(H)	δ(C)
1	2.05 (m)	23.6	2.52 (m)	18.6
	1.13 (m)		1.83 (m)	
2	2.04 (m)	25.1	2.06 (m)	25.5
	1.42 (m)		1.64 (m)	
3	4.19 (br.s)	74.1	4.64 (br.s)	74.5
4	1.94 (m)	35.2	2.27 (m)	37.0
	1.62 (m)		1.81 (m)	
5		73.5		73.8
6	1.93 (m)	35.9	2.17 (m)	36.0
	1.57 (m)		1.76 (m)	
7	1.51 (m)	21.4	1.52 (m)	24.8
	1.43 (m)		1.32 (m)	
8	1.96 (m)	41.4	2.26 (m)	41.9
9	1.41 (m)	39.0	1.64 (m)	39.5
10		54.3		55.2
11	2.26 (br.dd, *J* = 14.8, 3.6)	18.1	2.43 (m)	22.6
	1.65 (m)		1.40 (m)	
12	1.55 (m)	39.1	1.39 (m)	39.6
	1.23 (m)		1.28 (m)	
13		49.8		49.8
14		83.9		84.4
15	2.59 (dd, *J* = 15.6, 9.6)	40.0	2.02 (m)	32.2
	1.74 (m)		1.79 (m)	
16	5.41(ddd, *J* = 9.6, 5.8, 2.8)	73.7	2.07 (m)	27.2
			1.96 (m)	
17	3.15 (d, 8.4)	55.6	2.76 (m)	51.1
18	0.92 (s)	15.8	0.98 (s)	16.0
19	9.92 (s)	208.1	10.33 (s)	208.4
20		167.3		175.6
21	4.94 (dd, *J* = 18.4, 1.6)	75.5	5.25 (d, *J* = 18.4)	73.7
	4.83 (dd, *J* = 18.4, 1.6)		4.99 (d, *J* = 18.4)	
22	5.95 (dd, *J* = 1.6, 1.6)	121.5	6.10 (s)	117.8
23		173.9		174.4
AcO	1.93 (s)	170.4, 21.0		
	Dgn ^(a)^		Dgn	D-Dig
1′	4.50 (dd, *J* = 9.6, 2.4)	98.9	5.07 (br.d, *J* = 9.6)	99.5
2′	2.05 (m)	31.5	2.15 (m)	32.6
	1.66 (m)			
3′	3.31 (ddd, *J* = 12.8, 5.2, 2.0)	77.6	3.34 (br.dd, *J* = 12.0, 3.6)	79.7
4′	3.67 (br.s)	66.9	4.10 (br.s)	73.9
5′	3.43 (br.q, *J* = 6.8)	70.7	3.48 (br.q, *J* = 6.4)	71.1
6′	1.33 (d, *J* = 6.8)	16.7	1.49 (d, *J* = 6.4)	17.8
MeO	3.36 (s)	55.8	3.28 (s)	56.1
			Glc ^(b)^	
1′′			4.69 (d, *J* = 7.6)	104.9
2′′			3.91 (dd, *J* = 8.8, 7.6)	75.9
3′′			4.16 (dd, *J* = 8.8, 8.8)	78.5
4′′			4.10 (dd, *J* = 8.8, 8.8)	71.9
5′′			3.89 (m)	78.3
6′′			4.51 (dd, *J* = 11.2, 1.6)	63.1
			4.30 (dd, *J* = 11.2, 6.0)	

^(a)^ β-d-diginopyranose; ^(b)^ β-d-glucopyranose δ in ppm, *J* in Hz. Atom numbering as indicated in Figure 1.

All of the isolated cardenolides from *A. multiflora* were evaluated for cytotoxicity against six human cancer cell lines (HCT-116, HepG2, HeLa, SK-OV-3, SK-BR-3, and SK-MEL-5). As shown in Table 2, compounds **1**, **2**, **5**, and **6** showed significant inhibition activity against HCT-116, SK-OV-3, and SK-MEL-5 cell lines with IC_50_ values ranging from 0.06 ± 0.02 to 7.44 ± 1.98 µM. Compound **3** showed cytotoxic effects against the HeLa cell line with an IC_50_ value of 8.85 ± 0.39 µM. Compound **4** showed cytotoxicity against the SK-MEL-5 cell line with an IC_50_ value of 1.99 ± 0.28 µM.

**Table 2:** Cytotoxic activity of compounds **1**–**6** against human cancer cell lines (IC_50_ [µM] ^(a)^).

Compound	Cell lines (IC_50_) μM					
	HCT-116	HepG2	HeLa	SK-OV-3	SK-MEL-5	SK-BR-3
**1**	4.10 ± 0.38	14.65 ± 0.47	38.54 ± 1.08	2.34 ± 0.10	3.40 ± 0.67	38.35 ± 1.49
**2**	0.41 ± 0.13	17.99 ± 0.61	9.38 ± 0.15	0.06 ± 0.02	0.28 ± 0.06	2.58 ± 0.23
**3**	34.99 ± 1.39	30.12 ± 1.60	8.85 ± 0.39	25.38 ± 0.51	34.17 ± 1.78	80.38 ± 1.13
**4**	24.32 ± 1.26	26.61 ± 0.70	23.27 ± 1.73	41.02 ± 0.13	1.99 ± 0.28	23.94 ± 1.47
**5**	1.64 ± 0.13	2.87 ± 0.77	25.38 ± 0.15	0.76 ± 0.15	0.73 ± 0.14	5.10 ± 0.87
**6**	7.44 ± 1.98	13.71 ± 0.75	44.71 ± 0.89	4.63 ± 0.47	4.98 ± 0.56	21.30 ± 1.50
Doxorubicin	9.20 ±0.90	27.30 ± 0.50	3.20 ± 0.30	0.58 ± 0.08	4.80 ± 0.23	0.71 ± 0.05

^(a)^ All data were represented as mean ± SD of triplicate experiments.

## 3. Experimental Section

### 3.1. General

Column chromatography (CC): SiO_2_ (Kieselgel 60, Merck, Darmastdt, Germany) and ODS (LiChroprep RP-18, Merck) resins. TLC: Kieselgel 60 F_254_ and RP-18 F_254S_ (Merck) plates; visualization with UV lamp Spectroline Model ENF-240 C/F (Spectronics Corporation, Westbury, NY, USA) and spraying 10% H_2_SO_4_ soln. in MeOH and heating. Optical rotations: JASCO P-1010 digital polarimeter (Jasco, Tokyo, Japan). IR spectra: Perkin Elmer Spectrum One FT-IR spectrometer (Perkin Elmer, Beaconsfield, UK). FAB-MS: JEOL JMSAX-700 mass spectrometer (Jeol, Tokyo, Japan). NMR spectra: Varian Unity Inova AS-400 FT-NMR spectrometer (Varian, Palo Alto, CA, USA).

### 3.2. Plant Materials

*A. multiflora* Nishikawa & Koki Ito was supplied from the BMI Corporation (Uiwang, Korea) in January 2014, and was identified by *professor Dae-Keun Kim*, College of Pharmacy, Woosuk University, Jeonju, Korea. A voucher specimen (KHU2014-0117) has been reserved at the Laboratory of Natural Products Chemistry, Kyung Hee University, Yongin, Korea.

### 3.3. Extraction and Isolation

The whole plants of *A. multiflora* (1.5 kg) were extracted with 70% aqueous EtOH (30 L) at room temperature for 24 h. The concentrated EtOH extracts (106 g) were suspended in H_2_O (3 L) and then successively extracted with CH_2_Cl_2_ (AAC; 2.6 g), AcOEt (AAE; 0.7 g), BuOH (AAB; 12 g), and H_2_O (AAW; 89.2 g). The AAC (2.6 g) was subjected to CC [SiO_2_ (φ 4 × 11 cm); CH_2_Cl_2_/MeOH 18:1, 15:1, 7:1, 1.6 L of each] yielding 16 fractions, AAC-1–AAC-16. Fr. AAC-3 (200 mg, elution volume/total volume (V_e_/V_t_) 0.03–0.06) was subjected to CC [ODS (φ 3 × 7 cm); MeOH/H_2_O 3:1, 2.4 L], yielding 14 fractions, AAC-3-1–AAC-3-14. Fr. AAC-3-1 (52 mg, V_e_/V_t_ 0.00–0.09) was subjected to CC [SiO_2_ (φ 1.5 × 15 cm); Hexane/AcOEt 1:12, 0.5 L], yielding six fractions, AAC-3-1-1–AAC-3-1-6 along with a purified compound **1** [AAC-3-1-2; 12 mg; V_e_/V_t_ 0.46–0.52; TLC (ODS F_254S_; MeOH/H_2_O 5:2): *R_f_* 0.60]. Fr. AAC-4 (130 mg, V_e_/V_t_ 0.06–0.08) was subjected to CC [ODS (φ 3 × 7 cm); MeOH/H_2_O 4:5, 1.3 L], yielding 14 fractions, AAC-4-1–AAC-4-12 along with a purified compound **5** [AAC-4-9; 40 mg; V_e_/V_t_ 0.63–0.81; TLC (ODS F_254S_; MeOH/H_2_O 3:2): *R_f_* 0.45]. Fr. AAC-7 (200 mg, V_e_/V_t_ 0.17–0.22) was subjected to CC [ODS (φ 3 × 5 cm); MeOH/H_2_O 3:1, 1.6 l], yielding nine fractions, AAC-7-1–AAC-7-9 along with a purified compound **2** [AAC-7-2; 12 mg; V_e_/V_t_ 0.05–0.07; TLC (ODS F_254S_; MeOH/H_2_O 4:1): *R_f_* 0.45]. Fr. AAC-14 (121 mg, V_e_/V_t_ 0.62–0.66) was subjected to CC [ODS (φ 3 × 6 cm); MeOH/H_2_O 1:2, 2.7 L], yielding 11 fractions, AAC-14-1–AAC-14-11 along with a purified compound **3** [AAC-14-2; 8 mg; V_e_/V_t_ 0.04–0.19; TLC (ODS F_254S_; MeOH/H_2_O 2:1): *R_f_* 0.60], and compound **4** [AAC-14-6; 12 mg; V_e_/V_t_ 0.53–0.69; TLC (ODS F_254S_; MeOH/H_2_O 2:1): R_f_ 0.50]. The AAB (12 g) was subjected to CC [SiO_2_ (φ 7.5 × 16 cm); CH_2_Cl_2_/MeOH/H_2_O 13:3:1, 9:3:1, 7:3:1, 65:35:10, 7 L of each] yielding 15 fractions, AAB-1–AAC-15. Fr. AAC-5 (300 mg, V_e_/V_t_ 0.06–0.10) was subjected to CC [ODS (φ 2.5 × 5 cm); MeOH/H_2_O 2:3, 1 L], yielding nine fractions, AAB-5-1–AAC-5-9 along with a purified compound **6** [AAB-5-7; 28 mg; V_e_/V_t_ 0.38–0.71; TLC (ODS F_254S_; MeOH/H_2_O 6:5): *R_f_* 0.30].

### 3.4. Spectroscopic Data

*Adonioside A* (**1**)*.* White powder.

$${\left[\text{α}\right]}_{D}^{20}$$

= +23.9 (*c* = 0.5, MeOH). IR (CaF_2_): 3384, 2923, 1737, 1639, 1167, 1077 cm^−1^. ^1^H- and ^13^C-NMR: Table 1. negative HR-FAB-MS: 605.2971 ([M − H]^−^, C_32_H_45_O_11_; calc. 605.2962).

*Adonioside B* (**6**). White powder.

$${\left[\text{α}\right]}_{D}^{20}$$

= −94.4 (*c* = 0.7, pyridine). IR (CaF_2_): 3387, 2933, 1742, 1647, 1178, 1097 cm^−1^. ^1^H- and ^13^C-NMR: Table 1*.* positive HR-FAB-MS: 733.3511 ([M + Na]^+^, C_36_H_54_O_14_Na; calc. 733.3414).

### 3.5. Acid Hydrolysis of ***1*** and ***6***

Compound **1** (10 mg) and compound **6** (20 mg) were refluxed in 2 N HCl (0.3 mL) at 80 °C for 5 h, followed by neutralization with Ag_2_CO_3_ in excess and filtered through filter paper. The filtrate of **1** was subjected to CC [SiO_2_ (φ 1 × 10 cm); CHCl_3_/MeOH 12:1] to give fractions of sugar (**1a**) and aglycone, and that of **6** was subjected to CC [SiO_2_ (φ 1 × 10 cm); CHCl_3_/MeOH 12:1, 1:1] to give fractions of sugars **6a**, and **6b** and aglycone. The monosaccharides **1a**, **6a**, and **6b** in each sugar fraction were identified to be diginose, diginose, and glucose, respectively, by TLC comparison with authentic sugars. The *R_f_* values of diginose was 0.37 with CHCl_3_/MeOH 9:1 and 0.47 with CH_2_Cl_2_/EtOH 9:1, and that of glucose was 0.30 with CHCl_3_/MeOH/ H_2_O 7:3:0.5 [18,19].

### 3.6. Determination of Absolute Configuration of ***1a***, ***6a***, and ***6b***

The sugar fractions, **1a** (1 mg), **6a** (1.2 mg), and **6b** (1.2 mg), were measured for optical rotation values and compared with those reported in literature. Diginose, **1a** and **6a,** were determined to be d-form [**1a**: ${\left[\text{α}\right]}_{D}^{20}$ = +56.8 (*c* = 0.11, H_2_O), **6a**: ${\left[\text{α}\right]}_{D}^{20}$ = +55.5 (*c* = 0.12, H_2_O); d-diginose: ${\left[\text{α}\right]}_{D}^{20}$ = +59.6]. Glucose **6b** was determined to be d-form [**6b**: (${\left[\text{α}\right]}_{D}^{20}$ = +49.3 (*c* = 0.12, H_2_O); d-glucose: ${\left[\text{α}\right]}_{D}^{20}$ = +52.5] [16,17].

### 3.7. Cell Culture

Human hepatoma (HepG2), human cervix adenocarcinoma (HeLa), human ovarian adenocarcinoma (SK-OV-3), human breast adenocarcinoma (SK-BR-3), human colon carcinoma (HCT-116), human melanoma (SK-MEL-5) cells were obtained from the Korean Cell Line Bank (KCLB, Seoul, Korea). HepG2 and HeLa cells were maintained in Dulbecco’s modified Eagle’s medium (DMEM) supplemented with 10% (*v*/*v*) heat-inactivated fetal bovine serum (FBS) and 1% (*v*/*v*) penicillin-streptomycin in a humidified incubator with 5% CO_2_ at 37 °C. SK-OV-3, SK-BR-3, HCT-116, and SK-MEL-5 cells were maintained in RPMI1640 medium containing 10% (*v*/*v*) heat-inactivated FBS and 1% (*v*/*v*) penicillin-streptomycin in a humidified incubator with 5% CO_2_ at 37 °C. All cell culture media and reagents were purchased form Thermo Scientific Hyclone (Logan, UT, USA).

### 3.8. Cytotoxicity Assay

The cytotoxicity of cardenolides from *A. multiflora* was measured by a MTT colorimetric assay. Compounds were dissolved with dimethylsulfoxide (DMSO). The cells were seeded onto 96-well microplates at a density of 1 x 10^4^ cells per well in 100 μL of medium each. After incubation at 37 °C in a humidified incubator for 24 h, the cells were treated with various concentrations (1, 0.1, 0.5, 1, 5, 10, 50, 100 μM) of each compound in serum-free medium for 24 h. After incubation, 50 μL of MTT (5 mg/mL in PBS) was added to each well of the plate. The cells were incubated at 37 °C for 2 h. After removal from the medium, the cells were treated with 100 μL DMSO for 5 min and optical density measured using a microplate reader (BIO-TEK Inc., Winooski, VT, USA) at 550 nm. Cell viability was calculated as a percentage of viable cells in the compound-treated group *vs.* the control group by the following equation: Cell viability (%) = [OD (Compound) − OD (Blank)/OD (Control)-OD (Blank)] × 100.

### 3.9. Statistical Analysis

All experiments were performed with triplicate samples and repeated at least three times. The data are presented as means ± SD and statistical comparisons between groups were performed using 1-way ANOVA followed by Student’s *t*-test.

## 4. Conclusions

Two new and four known cardiac glycosides were isolated from the whole plants of *Adonis multiflora* Nishikawa & Koki Ito using open column chromatography and were identified based on spectroscopic data analysis, including NMR and FAB-MS. For the determination of absolute configuration, acid hydrolysis was performed. As a result, compound **1** and **6** were determined to be a 16-β-acetoxystrophanthidin 3-*O*-β-d-digonopyranoside, named adonioside A (**1**) and strophanthidin 3-*O*-β-d-diginopyranosyl-(1→4)-β-d-glucopyronoside, named adonioside B (**6**). In addition, the two new compounds **1** and **6** together with the two known compounds **2** and **5** showed significant cytotoxicity against six human cancer cell lines, HCT-116, HepG2, HeLa, SK-OV-3, and SK-MEL-5, and SK-BR-3, but we couldn’t establish a consistent structure-activity relationship. Consequently, these four compounds **1**, **2**, **5** and **6** merit futher *in vivo* study and on normal cell lines for bioactive selectivity. These findings suggest that *A. multiflora* may have potential be a useful therapeutic natural source for cancer prevention.

## Supplementary Materials

^1^H-NMR and ^13^C-NMR spectra of 1 and 6 and HMBC spectrum of 6 are available as supporting data. Supplementary materials can be accessed at: http://www.mdpi.com/1420-3049/20/11/19722/s1.
