# Supplementary material for: Two New Cyototoxic Cardenolides from the Whole Plants of Adonis multiflora Nishikawa & Koki Ito
Source: Molecules. 2015 Nov 23;20(11):20823–31. doi: 10.3390/molecules201119722 (PMC6332151; doi:10.3390/molecules201119722)

Supporting Information

In this 'Supporting Information' file for the manuscript "**Two new cyototoxic cardenolides from the whole plants of *Adonis multiflora* Nishikawa & Koki Ito**", ^{1}H-NMR, and ^{13}C-NMR spectra of two new compound **1** and **6** are available here as listed below.

Contents:

Page 2-4 ^{1}H and ^{13}C NMR of **1**

Page 5-7 ^{1}H ,^{13}C NMR and HMBC of **6**

^{1}H NMR of compound **1**.

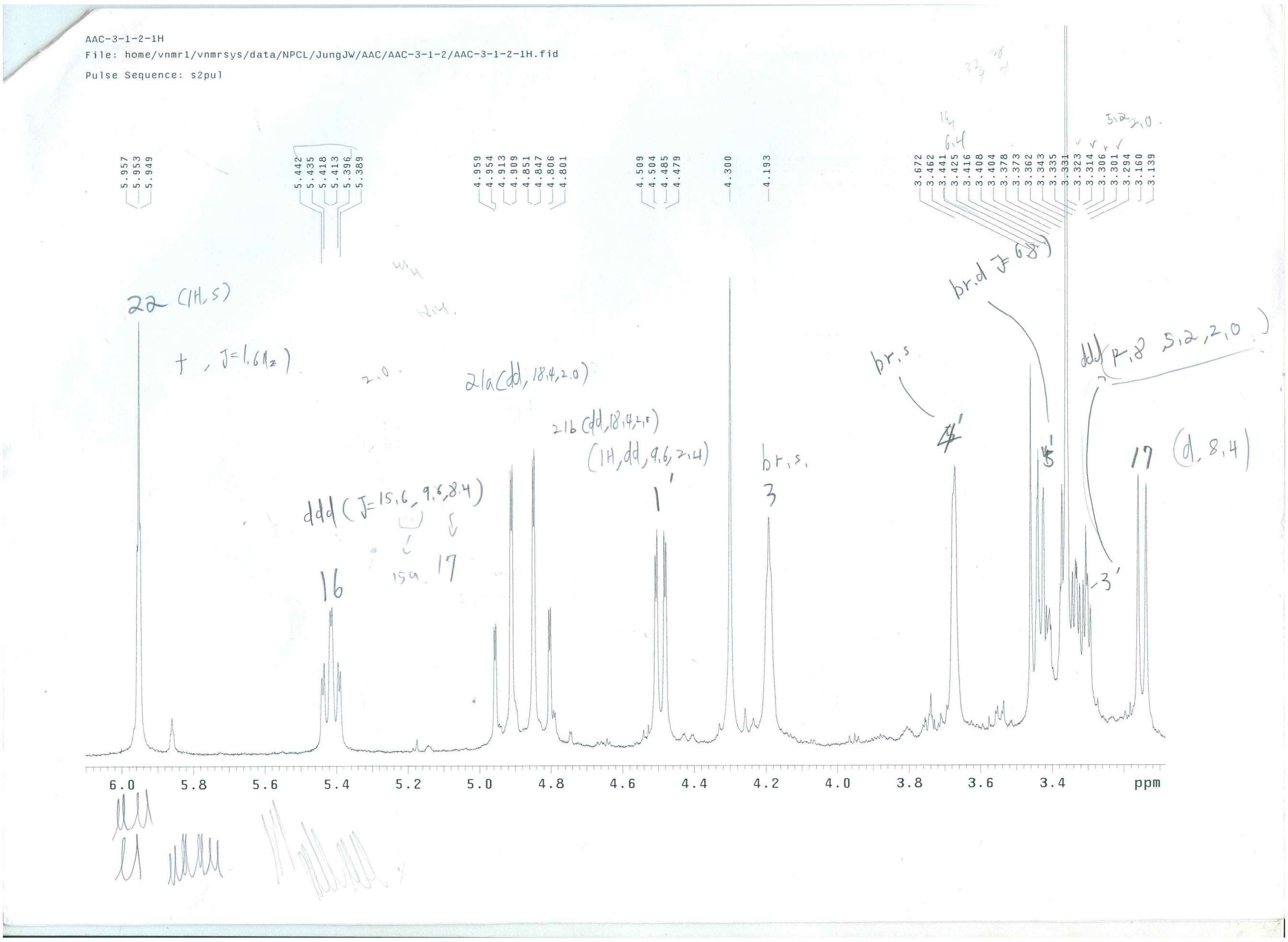

^{1}H NMR of compound **1**.

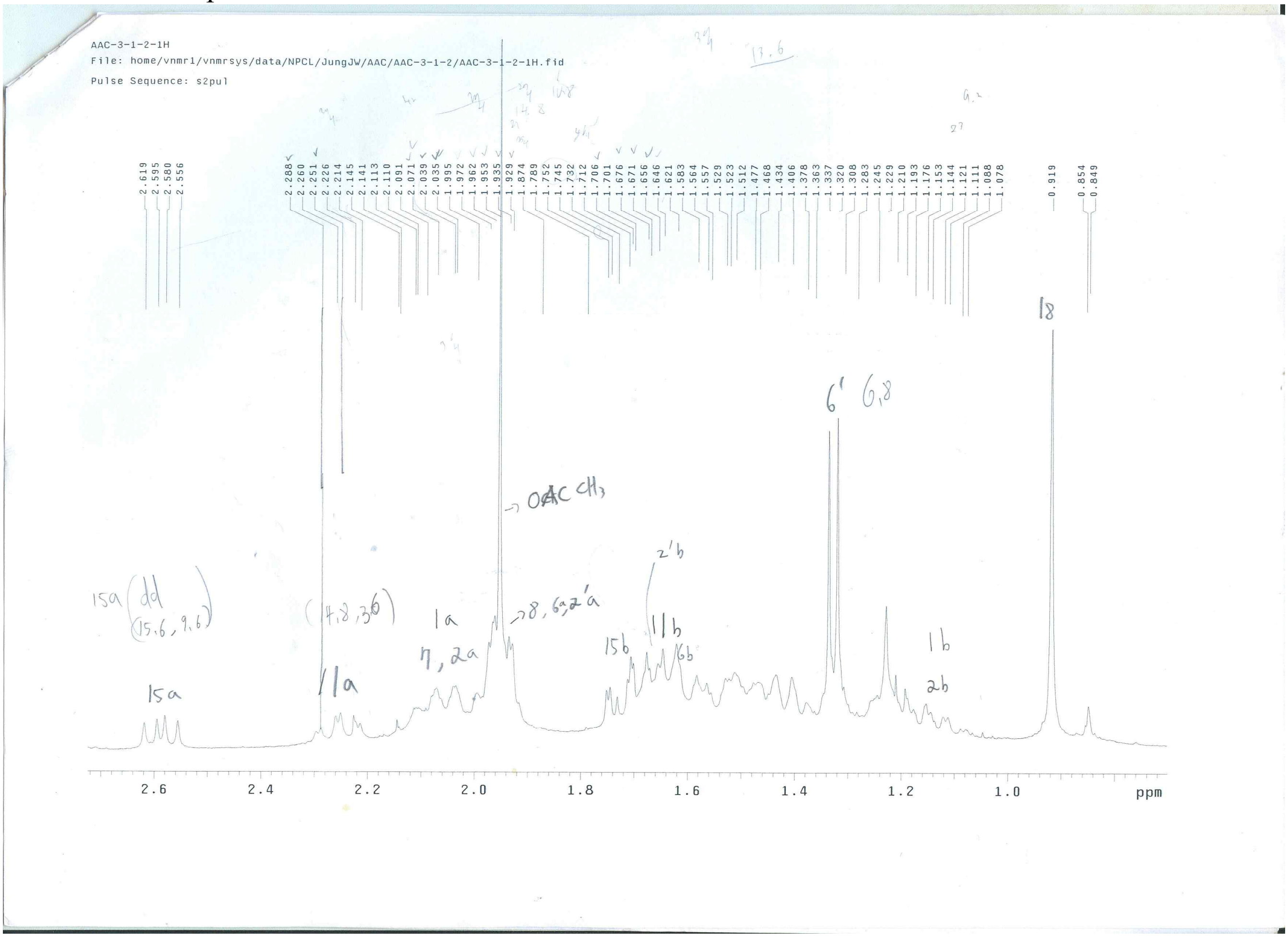

^{13}C NMR of compound **1**.

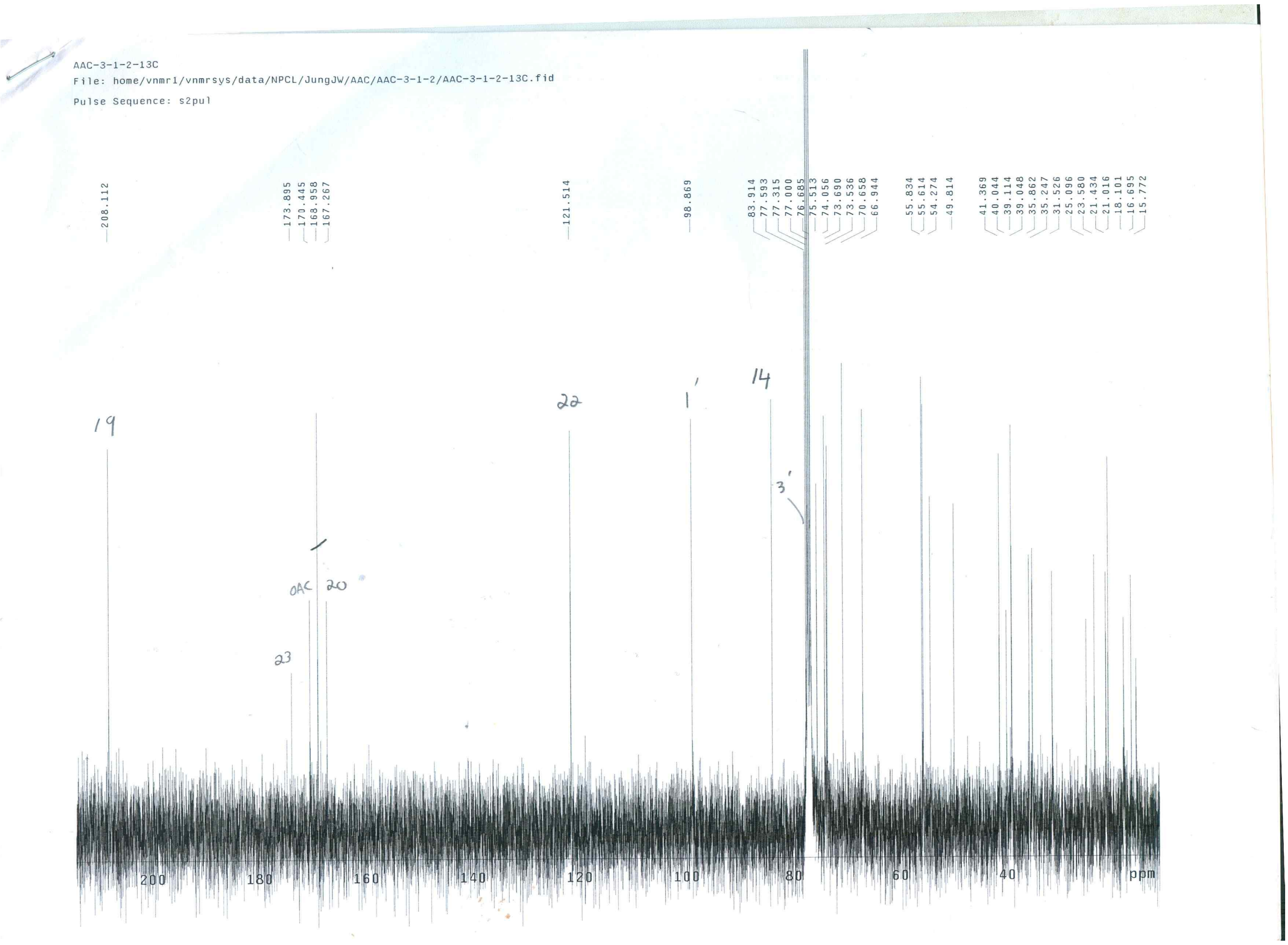

^{1}H NMR of compound **6**.

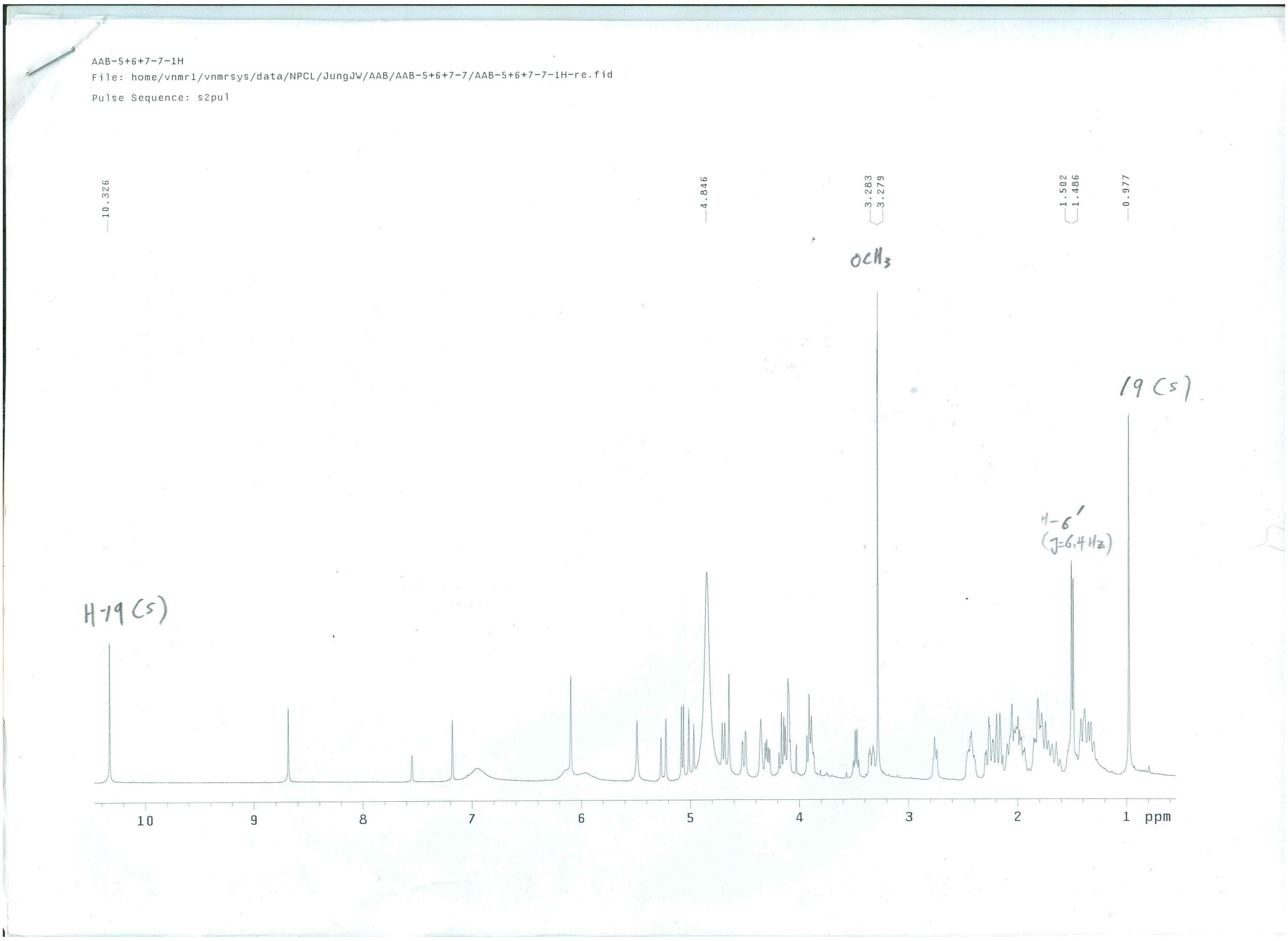

^{13}C NMR of compound **6**.

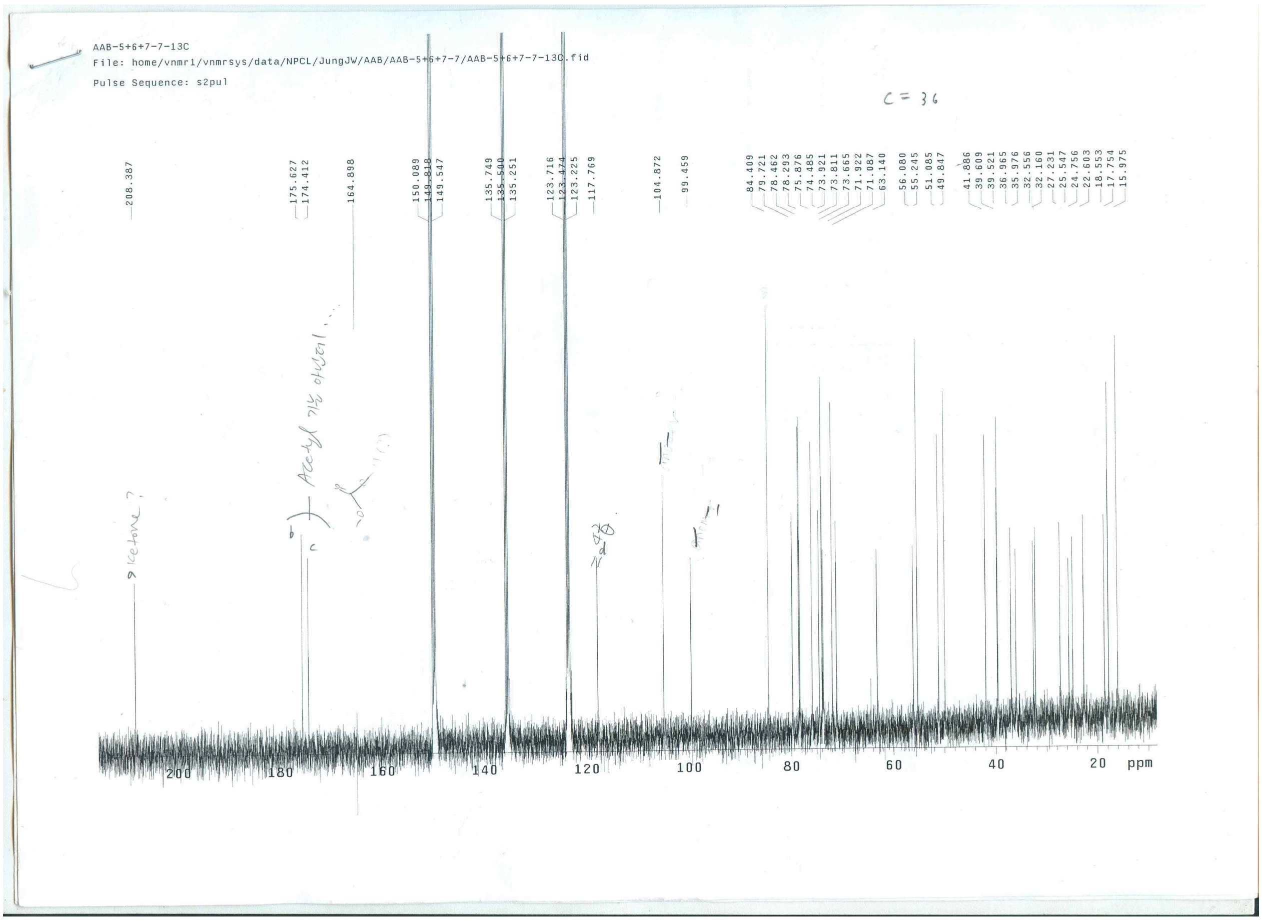

HMBC of compound **6**.

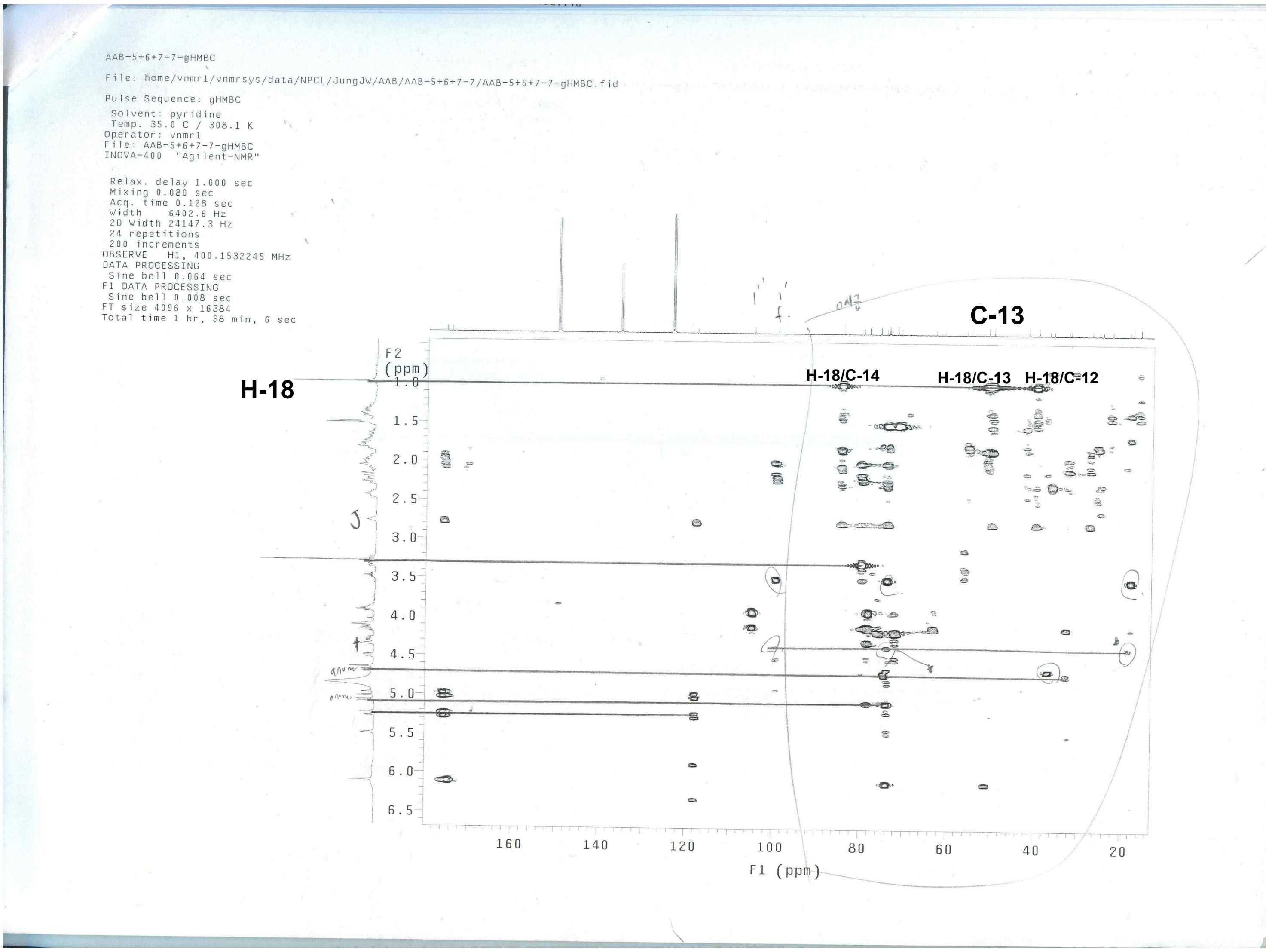

Supplement: Supplementary file 1 [file molecules-20-19722-s001.pdf]
